# Surgical treatment and adjuvant therapies of recurrent respiratory papillomatosis

**DOI:** 10.5935/1808-8694.20130114

**Published:** 2015-10-08

**Authors:** Melissa Ameloti Gomes Avelino, Tallyta Campos Domingues Teixeira Zaiden, Raquel Oliveira Gomes

**Affiliations:** a Post-doctoral Student, School of Medicine, Federal University of São Paulo (Adjunct Professor, Federal University of Goiás, Pontifical Catholic University of Goiás).; b Medical Student, School of Medicine, Pontifical Catholic University of Goiás (Medical Student, School of Medicine, Pontifical Catholic University of Goiás). Catholic University of Goiás.

**Keywords:** larynx, papilloma, papillomavirus infections, papillomavirus vaccines, therapeutics

## Abstract

Recurrent respiratory papillomatosis or recurrent laryngeal papillomatosis is a disease of the larynx caused by human papilloma virus, characterized by verrucous epithelial lesions and usually recurring. In the literature there are several types of treatment, such as surgery to cold, laser and/or use of microdebrider, as of adjuvant therapies; all possible to decrease the permanent sequelae of the disease.

**Objective:**

To review the literature regarding this disease with emphasis on surgical techniques and adjuvant therapies used today.

**Method:**

We used the literature review, through surveys based electronic data in the public domain, to search for articles between 1992-2012, using keywords: papilloma, human pappiloma virus infection, larynx, therapeutic, papilloma virus vaccine.

**Results:**

We surveyed 357 articles, of which 49 were used as the basis for this review. Scientific studies indicate a reduction of relapse in most adjuvant therapeutic presented. However, the survey showed different methodologies and samples, which did not allow to compare the types of treatment and adjuvant therapies.

**Conclusion:**

The choice of surgical technique varies among studies, but there is a trend to use the microdebrider. The newer adjuvant therapies, such as cidofovir, quadrivalent vaccine against human papilloma virus and bevacizumab, require further studies.

## INTRODUCTION

Recurrent respiratory papillomatosis (RRP) or laryngeal papillomatosis is a laryngeal disease caused by the human papilloma virus characterized by the proliferation of recurring epithelial wart-like lesions called papillomas. Papillomas commonly relapse and occur in significant numbers^1^, ^2^. RRP is the most frequently seen benign laryngeal tumor. Confluent papillomas introduce significant morbidity and may lead to dysphonia (voice alteration) and dyspnea (respiratory pattern alteration)^2^, ^3^.

RRP results from infection by the human papilloma virus (HPV). The most often detected virus subtypes are HPV-6 and HPV-11, seen in approximately 90% of the cases^1^, ^2^. HPV-16 and HPV-18 are found more rarely in children with RRP^3^, and have been more strongly correlated with malignant disease.

The mode of transmission of HPV has not been clearly established. Although transmission by sexual intercourse has been described, possible nonsexual routes include vertical and horizontal transmission, and autoinoculation. A recent meta-analysis showed that vertical transmission accounts for approximately 20% of the cases of HPV. However, horizontal transmission through saliva or other means has also been described^2^, ^4^. Upper airway exposure to HPV-6 and HPV-11 occurs somewhat frequently during one's life. It is likely that exposure to HPV is as common as exposure to other viruses, as studies have detected HPV DNA in the upper airways of as many as 25% of unaffected children and adults^5^.

According to Derkay^6^, some 1,500 to 2,500 new cases of RRP are diagnosed in the United States every year. Tasca et al.^7^ have reported an incidence of 6.9 cases for every 1,000 babies born from mothers with history of genital warts, against no cases per 1,000 births of children from mothers without history of genital warts. The authors have also reported that risk of RRP in children born from mothers with papillomas during pregnancy is 231.4 times higher than in mothers without history of papilloma. Incidence rates have been estimated at 4.3 cases for every 100,000 children and 1.8 case for every 100,000 adult subjects^6^.

The disease is usually categorized according to the severity of the signs and symptoms, and is divided into aggressive and non-aggressive RRP. Aggressive RRP is characterized by the need for ten or more surgical procedures, with three or more procedures being performed within the period of one year, or by when the disease extends distally toward the subglottic airway. Contrastingly, non-aggressive RRP is characterized by the need for fewer than ten procedures, with fewer than three procedures being performed within the period of one year, or no distal involvement reaching the subglottic area^8^.

Diagnosis is performed with the aid of fibroscopy (flexible scope) and laryngoscopy (rigid scope) and confirmation is attained through pathology testing. Macroscopically, laryngeal papillomas are pedunculated uneven nodular tumors of varying sizes. In terms of histology, papillomas are extremely vascularized, often keratinized tumors made of connective tissue covered by stratified squamous epithelium that project outwardly in finger-like fronds^9^.

Treatment is designed to maintain airway patency, improve patient voice quality, and prevent against complications. Surgery is the therapy of choice for RRP. Cold microsurgery, laser microsurgery, or surgery with a microdebrider are used to spare healthy tissue, prevent the formation of scar tissue, and remove all papillomas^1^, ^10^. Nonetheless, surgery cannot prevent recurrence, thus opening a precedent for the use of adjuvant therapies.

Adjuvant therapies have been given significant attention in the realm of RRP care. Many treatments have been described in the literature, but none appear to provide patients with a cure. Palliative care has been offered in the form of interferon-alpha, indole-3-carbinol, photodynamic therapy, cis-retinoic acid, cidofovir, aciclovir, ribavirin, bevacizumab, and tetravalent HPV vaccine^6^, ^7^, ^8^, ^9^, ^10^, ^11^, ^12^, ^13^, ^14^, ^15^, ^16^, ^17^, ^18^, ^19^.

There still is no consensus on the best course of adjuvant therapy. This study aimed to review the literature on RRP and focus on the most frequently used surgical approaches and adjuvant therapies.

## METHOD

The literature review was carried out based on the following question: ‘what are the surgical procedures and adjuvant therapies currently used in the treatment of recurrent respiratory papillomatosis?'. The search was carried out on databases Cochrane, LILACS (Literature in the Health Sciences in Latin America and the Caribbean), MedLine (USA National Library of Medicine), PubMed, and SciELO for papers written between January of 1992 and November of 2012. The following keywords were used in the search for papers: papilloma, papillomavirus infection, larynx, therapy, papillomavirus vaccines. Only papers written in Portuguese, Spanish, and English were included.

The papers found in the initial search were independently assessed by two authors. In order not to exclude important studies from the review, after a consensus meeting the two reviewers selected all potentially relevant papers accompanied or not by their abstracts. After the selection of relevant titles, papers in full were collected and assessed based on a protocol considering the following topics: type of study, sample, adopted approach, and reported results. All cross-sectional, longitudinal, prospective, and retrospective clinical trials, review papers, meta-analyses, case reports, and chapters published in Brazilian books meeting the criteria were included. Papers with significantly similar contents were excluded, and priority was given to the first authors to publish on the topic on Brazil or abroad, and/or to papers with larger and more recent samples. Included studies were chosen based on how well they answered the guiding question of this literature review. The book chapter selected for this review was chosen from a recent publication of 2011.

## RESULTS

Forty-nine of the 357 papers listed in the search result were included in this literature review. Most authors reported less recurrence with surgery and adjuvant therapy combined. However, the differences in materials and methods did not allow comparisons between types of surgical treatment and adjuvant therapies.

Authors agree that surgical removal of papillomas is the treatment of choice for patients with papillomatosis, despite differences between approaches. Larynx microsurgery can be performed with cold instruments, laser, and, as reported more recently, microdebriders. This review revealed that laryngeal sequelae were reported in 6% to 61% of the cases, depending on the chosen procedure. More recent studies have preferred microdebriders to other surgical instruments.

Drugs such as interferon, often described in the past, have not been used as adjuvant therapy in recent papers, indicating a trend toward the discontinuation of this type of therapy.

Although most recent papers still prefer cidofovir in the adjuvant therapy of RRP, some studies have failed to report significant improvements consequent to the use of this medication. In early studies, cidofovir was indicated only in local or intralesional treatment. Some authors have also described the use of inhaled cidofovir (Table 1).

**Table 1:** Results of studies on adjuvant therapy with cidofovir.

Authors	Year	Mode of administration	Results
Snoeck et al.	1998	Local injection	+
Pransk et al.	1999	Local injection	+
Pransk et al.	2000		+
Bielomowicz et al.	2002	Local injection	+
Pransky et al.	2003	Local injection	+
Lee et al.	2004	Local injection	+
Shirley et al.	2004	Local injection	-
Avelino et al.	2004	Local injection	+
Kimberlin	2004	Local injection	+
Pontes et al.	2006	Local injection	+
Naiman et al.	2006	Local injection	+
Naiman et al.	2006	Local injection	+
Dikkers	2006	Local injection	+
*Giles et al.	2006	Inhalation	+
Pudszhn et al.	2007	Local injection	+
Broekema et al.	2008	Local injection	+
**McMurray et al.	2008	Local injection	-
Pontes et al.	2009	Local injection	+
*Ksiazik et al.	2011	Inhalation	+
Carneiro et al.	2012	Local injection	+

* Case report;

** Randomized double-blind placebo-controlled study.

The papers written by Zeitels et al. as of 2009 have described very positive results for bevacizumab in association with KTP laser as an adjuvant therapy^14^, ^15^. However, the low number of studies on Avastin, the samples, and methods used in these studies do not allow conclusions to be drawn on the effectiveness of this RRP adjuvant therapy.

Although the HPV vaccine has been indicated to prevent against infection by the four subtypes of HPV (6, 11, 16, and 18), it has also been used as adjuvant therapy in patients with RRP. In 2008, Förster et al.^20^ suggested the vaccine was beneficial for RRP patients and called for the organization of a multicenter trial. In 2009, Pawlita & Gissmann^21^ recommended papillomatosis adjuvant therapy with tetravalent HPV vaccine. In 2011, Mudry et al.^17^ reported the case of a five-year-old submitted to multiple procedures for frequently recurring papillomas followed up for 17 months who entered remission after taking the HPV vaccine. However, this therapy has been described in the literature merely in the form of isolated case reports.

## DISCUSSION

Disease progress varies in RRP. While some patients with laryngeal papillomatosis see their condition regress spontaneously, others require multiple procedures for decades, with recurrence taking place within less than two weeks of surgery. Children usually experience more aggressive relapses and have less encouraging prognosis^1^, ^2^. The treatment of RRP can be a significant challenge in ENT practice. Although surgery is the treatment of choice, it does not prevent lesions from recurring.

Surgery aims to preserve uninvolved tissue, completely remove papillomas, and avoid scarring. It can be performed with cold instruments, CO_2_ laser, or microdebriders^1^, ^2^, ^10^. New approaches to surgery have been developed since cold laryngeal microsurgery. In the early 1970s, the advent of laser technology gave birth to a new surgical approach believed to offer the following advantages: better control over bleeding, higher accuracy, and longer disease-free periods. Thus, this approach was chosen as the first-line treatment for RRP^20^, ^21^, ^22^, ^23^, ^24^.

However, the indications and use of CO_2_ laser have been challenged in recent years by a number of authors. Studies have revealed the procedure presents higher risk of respiratory tract burns, and is more prone to producing stenosis and severe laryngeal scarring, distal injuries with tracheoesophageal fistulae, in addition to increasing the cost of treatment when compared to surgery with a microdebrider^2^, ^25^, ^26^. Other authors, such as Morgan e Crockett, reported complication rates of 61% and 36% with laser surgery, respectively^1^, ^2^. A retrospective study on the complications of CO_2_ laser surgery found that 10 of the 17 enrolled subjects had laryngeal scarring^27^. In 2007, Preuss et al.^12^ reported complication rates of only 6% in laser surgery against 20% when using the conventional approach.

In this context, microdebrider surgery has conquered adepts and is gradually replacing laser surgery as the surgical procedure of choice. Some authors have pointed out advantages such as shorter time of surgery, lower cost, lower risk of complications, and better patient voice quality when compared to CO_2_ laser^2^, ^12^, ^23^. The risk of sequelae is believed to vary depending on the type of procedure the patients are offered. Therefore, while some authors credit laser as the culprit of cases of laryngeal scarring, others prefer to defend its use. Nonetheless, regardless of the approach/instruments used, the virus remains latent even after careful resection, thus leading to recurrence^1^. Managing recurrence often requires extensive manipulation of the larynx, and possibly leads to permanent damage in the form of stenosis, anterior and posterior membrane neoformation, vocal fold injury, and granulation tissue formation, among others^2^. A retrospective study with 50 children with RRP revealed that laryngeal sequelae occur frequently, particularly anterior commissure synechia and glottic stenosis^1^.

Adjuvant therapies gained ground in medical practice as an attempt to minimize recurrence, its complications, the negative impacts it has upon patients' lives, and reduce or eliminate the need for future surgery. The most widely accepted criteria to start adjuvant therapy are: need for more than four surgical procedures per year, lesions extending toward the subglottic region, and short disease-free intervals leading to airway involvement^28^. Between 12.6% and 47.6% of the children with RRP are given adjuvant therapy^7^, ^10^, ^18^, ^29^, as recommended in cases of aggressive disease with frequently recurring papillomas or distal involvement of the airways^10^, ^30^.

Interferon-alpha was the first medication offered as adjuvant therapy to RRP patients. Although it is still used today, interferon-alpha is no longer as popular as it once was. In its early days, the drug seemed to produce good outcomes, particularly within the first few months of use, but patients experienced multiple side effects. Other therapies and drugs such as indole-3-carbinol, photodynamic therapy, and cis-retinoic acid were studied, but none grew to become the therapy of choice for subjects with RRP^3^, ^10^.

In recent years, antiviral drug cidofovir [(S)-1-[(3-hydroxy-2-phosphonyl methoxy) propyl] cytosine (HPMPC)] was introduced as a potent replication inhibitor for HPV, adenovirus, cytomegalovirus, and herpesvirus^2^, ^10^, ^31^, ^32^, ^33^, ^34^, ^35^, ^36^, ^37^, ^38^, ^39^, ^40^, ^41^.

Cidofovir has been used as an adjuvant drug since 1998. In 1998, Snoeck et al. studied the use of cidofovir injections to control RRP in adult subjects, while Pransky et al. analyzed its impact on pediatric populations in 1999 and 2000^31^, ^32^, ^33^. The drug is a cytosine analog and a potent inhibitor of viral replication. Nephrotoxicity is a significant, frequently seen side effect described in patients on cidofovir. Other adverse effects include neutropenia, weakness, nausea, and diarrhea^10^. However, a study on the local administration of cidofovir to treat children with RRP did not report alterations in their lab workup or adverse effects to the medication, apparently reaffirming the drug's safety when administered locally^3^, ^7^, ^32^, ^33^, ^34^, ^35^, ^36^, ^37^, ^38^, ^39^. The literature offers a vast array of papers on cidofovir. However, favorable outcomes (remission or less severe disease in most patients)^33^, ^34^, ^35^, ^36^, ^37^, ^38^, ^39^ have been reported along with no significant improvements in disease recurrence^40^, ^41^.

Cidofovir was described as the most used adjuvant therapy on a survey carried out with members of the American Society of Pediatric Otolaryngology, with interferon ranking second. Sixty-one percent of the patients followed up in the services of the physicians included in the survey improved or were disease-free after treatment, against only four percent of cases in which the disease became more severe. Nonetheless, this study may contain bias and sampling errors, in addition to having used a subjective assessment tool to analyze patient responses, which may have affected the published results^18^.

A study carried out by members of the British Association of Paediatric Otorhinolaryngology in 2006 showed that cidofovir was the most frequently prescribed drug in the treatment of individuals with RRP. However, the study failed to determine the role of cidofovir in the treatment or to quantify decreases in recurrence^7^. Some authors began to wonder whether intralesional cidofovir would increase the potential for the development of malignant disease. The Food and Drug Administration (FDA) has considered cidofovir to be possibly carcinogenic, once it increased the incidence of breast adenocarcinoma in rats^42^. Intralesional cidofovir has also been connected to cases of dysplasia in human beings^43^, ^44^, ^45^. Although Broekema reported malignant degeneration in a group of patients given 2.7% cidofovir, the author pointed out that spontaneous malignant degeneration occurs in 2-3% of RRP patients, thus ruling out the alleged association between cidofovir and increased incidence of malignant disease. All the more, HPV increases the chance of development of malignant disease alone, independently from cidofovir^43^.

In 2008, Lindsay et al.^13^ analyzed the histology of papillomas before and after the administration of cidofovir and did not observe the occurrence of dysplasia in the patients. The author also reported that cidofovir could potentially cause vocal fold scarring, and suggested the drug should be used with caution on the vocal folds^13^.

Only case reports have described the use of inhaled cidofovir in the literature. In 2006, a report was published in which inhaled cidofovir was used to reach lung lesions^43^, ^46^. It described the case of an eleven-month-old girl with respiratory failure and stridor (due to RRP) who underwent a tracheostomy. In order to reach papillomas in the lungs, cidofovir was administered with the aid of a nebulizer connected to the tracheostomy tube. The only reported complication was hemoptysis, which resolved as the drug dosage was reduced. The patient responded well, and her lower airway lesions shrank to a size invisible to the naked eye and the complications disappeared^46^.

In 2011, another report described the case of a previously healthy four-month-old with weak cry, hoarseness, and increased respiratory effort. Examination with a flexible fiberscope revealed the patient had papillomas in the glottis, which were removed with a microdebrider. Despite treatment with a microdebrider every two weeks, intralesional cidofovir, and systemic interferon-alpha, the patient's health deteriorated substantially from persistent complications. The patient was then offered inhaled cidofovir after the failure of conventional therapies. Significant improvement was seen after six weeks of treatment. Nonetheless, further studies are needed to look into the long-term effectiveness of the treatment and the safety profile of inhaled cidofovir^47^.

According to this literature review, although cidofovir is the most frequently used adjuvant therapy, it is not universally accepted. The need to find alternative RRP adjuvant therapies has recently led to the development of new treatment modes with bevacizumab and tetravalent HPV vaccine.

Considering the use of bevacizumab (Avastin), this review showed that vascularization is believed to be a determining factor in how quickly papillomas reappear. Thus, bevacizumab would work as an angiogenesis inhibitor, to hamper or stop the growth of papillomas and associated complications^15^, ^16^. Complementary modes of action and reported safety have led authors to advocate the combination of local injections of bevacizumab and KTP laser surgery. The clinical successes of bevacizumab and KTP laser reported within the last two years and the lack of complications after over 200 laryngeal injections of bevacizumab have pushed this therapy to the top of the list of RRP treatment options^15^, ^16^. However, larger and more significant studies are required to prove the benefits of this therapy. Relevant questions have been posed about the combined use of bevacizumab and KTP laser. Is activation by laser needed for bevacizumab to yield proper benefit? Could the drug be used without KTP laser?

Tetravalent HPV vaccine protects against viral subtypes 6, 11, 16, and 18 and works on an acquired immunity mechanism characterized by specificity and memory; it is mediated by B-cells, T-cells, antibodies, and cytokines^48^, ^49^. When an antigen gets in touch with these cells, a specific response is produced. Then, the antigen produces a strong qualitative and quantitative response, with high levels of antibodies, thus configuring the immune memory and long-term protection. Immunogenicity, the ability to provoke a specific immune response, is usually measured as a function of antibody counts^48^. Tetravalent HPV vaccine is a killed vaccine, which does not contain antigens able to replicate in the individuals given the shot. It is produced using molecular biology techniques. Tetravalent HPV vaccine contains particles produced in vitro from viral proteins which are similar to the virus. They combine spontaneously to form structures similar to the whole virus^48^, ^49^. It is important to note that the vaccine does not have infectious or oncogenic potential, and that it is very safe. Clinical trials currently in progress have not reported significant adverse events; side effects were limited to pain, redness, and edema on the site of injection, low fever, headache, and syncope^48^.

Although vaccination is currently considered only for patients not infected by HPV, it has also been studied for patients with RRP. In 2008, Förster reported the case of a two-year-old with aggressive laryngeal papillomatosis. After three shots of tetravalent HPV injection, the patient's condition was stabilized without surgery 10 months into follow-up^2^, ^20^.

In 2009, Pawlita & Gissmann described the use of tetravalent HPV vaccine as an adjuvant therapy for papillomatosis, reinforcing the role this medication could have in infected patients and the very low risks associated with its use^2^, ^21^.

In 2011, Mudry et al. reported the case of a five-year-old with frequent recurring papillomas submitted to multiple procedures. The patient entered remission after the administration of tetravalent HPV vaccine. The 17 months for which this patient has been disease-free ranks as one of the longest periods without disease reported in the literature^2^, ^17^.

Many case report authors have described changes to the natural course of the disease, stabilization, or significant reductions in papilloma recurrence after vaccination. However, as these are only isolated case reports, comprehensive multicenter trials are required to assess the true benefits of vaccination in the treatment of RRP^16^, ^17^, ^20^, ^21^.

This literature review indicated that the use of surgery and adjuvant therapies largely depends on the context of each service, the experience of the physicians involved, the possible adverse effects and, mainly, the acceptance of patients, and not only on how effective the treatment can be.

## CONCLUSION

This literature review showed there is no consensus around one surgical approach for RRP, despite a recent trend toward the use of microdebriders. In regards to adjuvant therapies, although cidofovir is the most frequently used drug, large multicenter trials are required to better describe the levels of efficacy offered by this drug.
